# A New Mouse Model That Spontaneously Develops Chronic Liver Inflammation and Fibrosis

**DOI:** 10.1371/journal.pone.0159850

**Published:** 2016-07-21

**Authors:** Nina Fransén-Pettersson, Nadia Duarte, Julia Nilsson, Marie Lundholm, Sofia Mayans, Åsa Larefalk, Tine D. Hannibal, Lisbeth Hansen, Anja Schmidt-Christensen, Fredrik Ivars, Susanna Cardell, Richard Palmqvist, Björn Rozell, Dan Holmberg

**Affiliations:** 1 EMV Immunology, BMC, Lund University, 221 00 Lund, Sweden; 2 ISIM- Immunology, Faculty of Health and Medical Sciences, Copenhagen University, 2200 Copenhagen, Denmark; 3 Department of Medical Bioscience-Medical Genetics, Umeå University, 901 85 Umeå, Sweden; 4 Instituto Gulbenkian de Sciencia, Oeiras, 2780–156 Oeiras, Portugal; 5 Institute for Biomedicine-Infection and Immunology, University of Gothenburg, 405 30, Göteborg, Sweden; 6 Department of Medical Bioscience-Pathology, Faculty of Medicine, Umeå University, 901 85 Umeå, Sweden; 7 Faculty of Health and Medical Sciences, Copenhagen University, 2200 Copenhagen, Denmark; University of Navarra School of Medicine and Center for Applied Medical Research (CIMA), SPAIN

## Abstract

Here we characterize a new animal model that spontaneously develops chronic inflammation and fibrosis in multiple organs, the non-obese diabetic inflammation and fibrosis (N-IF) mouse. In the liver, the N-IF mouse displays inflammation and fibrosis particularly evident around portal tracts and central veins and accompanied with evidence of abnormal intrahepatic bile ducts. The extensive cellular infiltration consists mainly of macrophages, granulocytes, particularly eosinophils, and mast cells. This inflammatory syndrome is mediated by a transgenic population of natural killer T cells (NKT) induced in an immunodeficient NOD genetic background. The disease is transferrable to immunodeficient recipients, while polyclonal T cells from unaffected syngeneic donors can inhibit the disease phenotype. Because of the fibrotic component, early on-set, spontaneous nature and reproducibility, this novel mouse model provides a unique tool to gain further insight into the underlying mechanisms mediating transformation of chronic inflammation into fibrosis and to evaluate intervention protocols for treating conditions of fibrotic disorders.

## Introduction

Formation and remodeling of the extracellular matrix is critical during wound healing and scar formation but excessive connective tissue formation, as seen in fibrosis, can be detrimental and lead to organ failure [1]. Local and systemic chronic inflammatory conditions triggered by trauma, toxic damage, metabolic dysregulation, persistent infection or autoimmune reactions are typically accompanying fibrosis development. Although fibrotic disease conditions may have substantially different triggering factors and etiology it is generally thought that key components of the pathogenesis are shared [2, 3]. In this perspective, fibrosis could be regarded as a pathologic process with overlapping pathogenic factors.

The liver is one of the organs where fibrosis constitutes a serious clinical problem as most chronic liver diseases are associated with liver fibrosis. In order to better understand the development of liver fibrosis, several spontaneous as well as induced animal models have been established and have constituted important tools for analysis of different aspects of the disease process [4, 5]. Despite these efforts, there are still no efficient anti-fibrotic treatments available to date. A major limitation in the attempts to understand the underlying mechanisms and to establish efficient anti-fibrotic treatment protocols has been the restricted set of suitable animal models. One caveat has been the difficulty in reproducing the process of persistent inflammation leading to tissue remodeling and fibrosis that is commonly observed in human fibrotic disorders [3]. Thus additional animal models representing different aspects of fibrosis development are needed.

While myeloid cells are of great importance in conducting and maintaining an inflammatory response, substantial data supports a prominent role for different T cell subsets in the development and control of fibrosis [6–8]. However, the specific role of different T cell subsets remains unclear.

Natural killer T (NKT) cells constitute a population of unconventional T lymphocytes that express the αβ T cell receptor (TCR) together with several NK surface markers and recognize glycolipids presented by the MHC class I like CD1d molecule [9, 10]. The NKT cell population is heterogeneous where the majority, referred to as type I NKT cells, express an invariant TCR and display specificity for glycolipids presented by CD1d, with the prototype antigen being α-GalCer. Type II NKT cells resemble type I NKT cells in their restriction to CD1d, but use a diverse set of TCR and have a less well-defined range of antigen specificities. NKT cells are highly enriched in the liver and have been shown to be able to promote as well as to protect from inflammation and fibrosis development, suggesting that the net effect of the NKT cells depends on the balance between these properties [11–14]. In line with this activated NKT cells are known to be able to produce large amounts of both anti-fibrotic (e.g. interferon (IFN)-γ) and profibrotic (e.g. interleukin-4 (IL-4), IL-13) cytokines [12, 13, 15].

In this report, we present the phenotypic characteristics of a new animal model, the N-IF mouse that spontaneously develops inflammation-driven fibrosis affecting the liver as well as other organs. The disease of the N-IF mouse is driven by transgenic type II NKT cells generated in immunodeficient NOD.Rag2^-/-^ mice by expressing a transgenic α,β T cell receptor isolated from an type II NKT cell line. The disease phenotype can be transferred with splenocytes to naïve NOD.Rag2^-/-^ mice, and can be inhibited by a polyclonal T cell population derived from unaffected NOD mice. This novel mouse model will provide insight into the mechanisms controlling the progression of chronic inflammation to a fibrotic process and constitutes a potential tool for improved effect tests of therapy strategies specifically addressing the fibrosis process.

## Material and Methods

### Ethics statement

Animal experiments were performed in strict accordance with the recommendations for the use of laboratory animals from the Swedish board of agriculture. The ethics committees of Umeå University and Lund University approved all animal experimental procedures (Permit number: M243-14). Mice were sacrificed by cervical dislocation. All efforts were made to minimize suffering.

### Mice

All mice were bred and maintained in a specific pathogen-free facility at Umeå University or at the animal facility at Lund University. 24αβNOD and NOD.Rag2^-/-^ mice have been described before [16, 17]. The N-IF mouse was generated by crossing the 24αβNOD and the NOD.Rag2^-/-^ mouse strains. The 24αβB6.Rag2^-/-^ mouse strain was generated by backcrossing the N-IF mouse with the B6.Rag2^-/-^ mouse strains for 10 generations.

### Histology

Liver tissue were fixed in 4% neutral buffered formalin, embedded in paraffin and sectioned. Sections (5 μm) were stained with hematoxylin and eosin (H&E), Toluidine blue (TolB) or Sirius red and were evaluated microscopically. Immunohistochemical staining was performed on liver biopsies fixed in 4% paraformaldehyde and embedded in OCT. The frozen tissues were cut in 5 μm thick sections and stained using primary antibody against cytokeratin 7 (1:1500, Abcam, EPR17078), F4/80 (1:200, AbD Serotec, CI:A3-1), CD3 (1:200, Sigma C7930), Collagen I (1:200, Abcam, ab21286) CD45 (1:200, eBioscience, 30-F11), matrix metallopeptidase 9 (MMP9) (1:300, Abcam, ab38898) and anti-smooth muscle actin (ASMA) (1:100, Abcam, ab5694) and secondary anti-rabbit (1:2000, Alexa 594), anti-rat (1:2000, Alexa 647) antibodies. The nuclei were visualized with DAPI. The sections where analyzed using confocal microscopy.

### Serum parameters and hydroxyproline examination

Serum was collected by centrifugation of clotted whole blood for 10 minutes at 1500 x g. The cleared supernatant was collected and sent to The University Animal Hospital, SLU, Uppsala for measuring AST, ALT, ALP, total bilirubin and bile acid using a fully automated Architect c4000 (Abbott Laboratories, Abbott Park, IL, US). The liver hydroxyproline content was determined with the *Hydroxyproline Colorimetric Assay kit* (BioVision) according to manufacturer´s instructions.

### Flow cytometry analysis

Liver leukocytes were obtained by incubating cut pieces of liver in 1.0 mg/ml collagenase II solution (Sigma) for 40 min at 37°C, after which the tissue was minced through a 70 μm mesh and leukocytes were separated on a 50/25 Percoll (GE Healthcare) by centrifugation. Cells were stained in FACS buffer (3% FCS in PBS). Prior to surface staining the cells were incubated with the 2.4G2 (anti-CD16/CD32) Ab (BD Biosciences), to prevent unspecific binding. The cells were then incubated with fluorochrome-conjugated anti-murine antibodies specific for the following cell surface markers: CD45 (30-F11) and Ly6G (1A8) from Biolegend, CD11b (M1/70), Vα3.2 (RR3-16) and Vβ9 (MR10-2) from eBioscience and Siglec-F (E50-2440) from BD Bioscience. Cell viability was determined using fixable viability dye (eBioscience). The stained cells were analyzed using a BD LSR II flow cytometer and Kaluza software (Beckman Coulter). For gating strategy see S1 Fig.

### Cell activation and cytokine analysis

Single-cell suspensions from spleen were prepared by disrupting the tissue through a 70 μm mesh. Total splenocytes (2x10^6^) and liver leukocytes (2x10^5^) were activated using anti-CD3 Ab (4μg/ml, clone 154-2C11, BD Biosciences). Sorted (>94% purity) Vα3.2^+^/Vβ9^+^ NKT cells from liver (1x10^5^) were activated by anti-CD3/CD28 dynabeads (1:1 ratio bead:cell, Life Technologies). In all cases cells were grown in complete medium (RPMI 1640 medium supplemented with 10% FCS, 100 U/mL penicillin/streptomycin, 2.5% sodium bicarbonate (7.5% solution), 1 mM sodium pyruvate and 69 μM 1-thioglycerol). The supernatants were collected after 24h and analyzed for cytokines using the mouse Th1/Th2/Th17/Th22 13-plex (eBiosciences) according to manufacturer´s instructions.

### Adoptive transfer experiment

Splenocytes from 8–12 weeks old donor N-IF mice were processed to single cell suspension and 25x10^6^ total spleen cells were transferred *i*.*v*. to 5–6 weeks old NOD.Rag2^-/-^ recipient mice. Alternatively, splenocytes from donor N-IF mice were depleted of the majority of transgenic NKT cells (more than 85% of cells using MACS sorting with biotinylated anti-Vα3.2 antibodies (RR3-16, BD Biosciences) and streptavidin-conjugated microbeads (Miltenyi Biotec). 24x10^6^ splenocytes depleted of Vα3.2^+^ (-NKT) were injected *i*.*v*. to NOD.Rag2^-/-^ recipient mice. For analysis of the effect of normal splenocytes on the N-IF phenotype, 25x10^6^ total splenocytes from 8–12 weeks old NOD mice were transferred *i*.*v*. to 5–6 weeks old N-IF recipient mice. Alternatively, NOD splenocytes enriched for or depleted of T cells were transferred *i*.*v*. to 5–6 weeks old N-IF recipient mice after MACS sorting using the pan-T cell microbead kit (Miltenyi Biotec) according to manufactures instruction. 10x10^6^ T cell enriched splenocytes or 15x10^6^ T cell depleted splenocytes, reflecting the ratio of these cell subsets in the spleen, were injected *i*.*v*. to 5–6 weeks old N-IF recipient mice. All recipients were observed daily after adoptive transfer and analyzed 5 weeks after transfer.

### Statistical analysis

Statistical results are presented as mean and standard error of mean (SEM). Statistical analysis was performed using the GraphPad Prism 5 software and differences between two groups were considered significant when p < 0.05 as assessed by unpaired, two-tailed Student’s *t* test.

## Results

### Generation of the N-IF mouse model for inflammation and fibrosis

**Fig 1:**
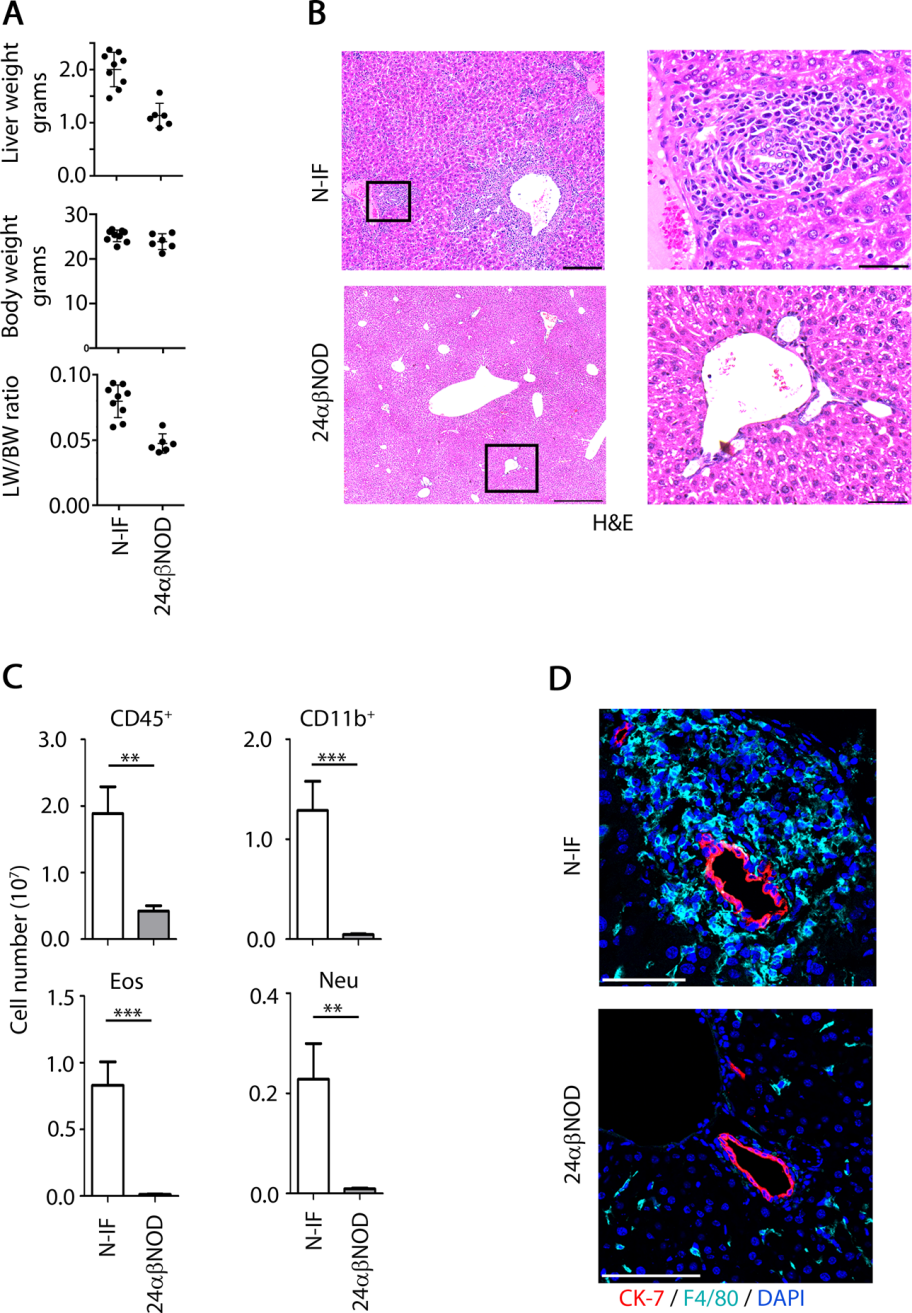
Hepatic inflammation and abnormal intrahepatic bile ducts in the N-IF mouse. (A) Liver weight (LW), body weight (BW) and LW/BW ratio of 8 weeks old, female N-IF mice (left, n = 9) and of aged matched female 24αβNOD control mice (right, n = 5); (B) H&E stained liver sections from 10–12 weeks old, female N-IF and 24αβNOD control mice. Squared areas are highlighted. Images shown are representative images from three independent experiments with a total of eight mice. Scale bars are 100 μm in the overview images (left) and 50 μm in the highlighted images (right). (C) Flow cytometry analysis of viable liver leukocytes from N-IF and 24αβNOD mice (n = 9). Number of leukocyte (CD45^+^), CD11b^+^ cells gated from viable CD45^+^ cells and eosinophils (Eos) and neutrophils (Neu) gated from the CD11b^+^ population and defined as being SiglecF^+^ and Ly6G^+^ respectively. Data are pooled from three independent experiments and shown as mean ± SEM. **p<0.01 and ***p<0.001 by unpaired *t*-test. (D) Immunofluorescence staining of bile ducts (CK-7, red), macrophages (F4/80, light blue) and nuclei (DAPI, blue) in livers of 8 week old N-IF and 24αβNOD control mice. Representative images from two independent experiments with a total of six mice are shown. Scale bars are 100 μm.

The 24αβNOD mouse overproducing a monoclonal NKT cell population was previously generated and found to develop diabetes with a significantly reduced incidence compared to wild type NOD mice [16]. Unexpectedly, we found that the offspring produced by crossing the 24αβNOD mouse with a NOD.Rag2^-/-^ mouse to generate 24αβNOD.Rag2^-/-^ (here denoted N-IF) mice, developed an inflammatory syndrome most evident in the liver but also affecting other organ systems such as skin, and kidney. In the liver, a hepatomegaly was observed in N-IF mice (S2A Fig) with a 100% penetrance. This was evidenced from the increasing liver weight (LW) to body weight (BW) ratio of the N-IF mice compared to controls (Fig 1A). Histologically we observed extensive cellular infiltration dominated by granulocytes, particularly eosinophils, macrophages, mast cells and multinucleated giant cells (Fig 1 and S2 Fig), which could be observed already at 3 weeks of age (S3 Fig). The cellular composition was also confirmed and quantified using flow cytometry analysis of liver leukocytes (Fig 1C). In addition, scattered megakaryocytes and hepatic extramedullary hematopoiesis with colonies of myelopoiesis showing both neutrophilic and eosiniphilic differentiation was observed in the N-IF mouse liver (S2C and S2D Fig).

The granulomatous inflammation in the N-IF mouse was most pronounced in the portal tracts and central veins associated with abnormal intrahepatic bile ducts (Fig 1B and 1D). An observed increase in the serum levels of bile acid in the N-IF mouse compared to control strains suggested that the N-IF mouse developed cholestasis (S1 Table), however no increase in serum bilirubin and a decrease rather than an increase in alkaline phosphatase was detected (S1 Table).

**Fig 2:**
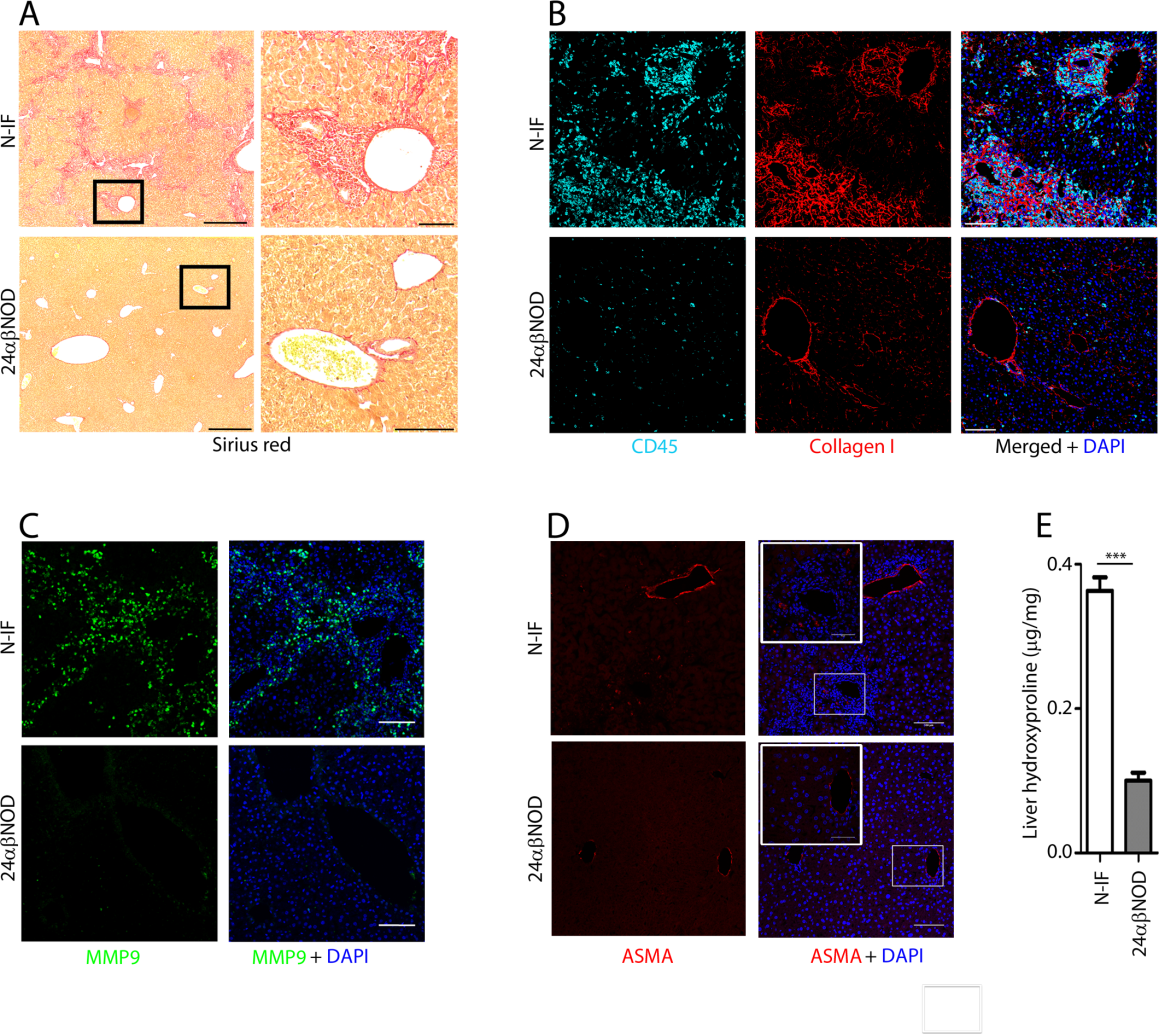
The N-IF mouse develops liver fibrosis. Representative liver sections from 11 weeks old N-IF and 24αβNOD control mice stained with Sirius red. Representative images from two independent experiments with a total of six mice are shown. Scale bars are 500 μm in the overview images (left) and 100 μm in the enlarged images (right) (A). Immunofluorescence staining showing (B) collagen I (red), CD45 (cyan) and DAPI (blue); (C) MMP9 (green) and DAPI (blue) and; (D) ASMA (red) and DAPI (blue), in 12 weeks old N-IF and 24αβNOD control mouse livers. Representative images from two independent experiments with a total of six mice are shown. Scale bars are 100 μm. (E) Hydroxyproline content per mg liver tissue from N-IF and 24αβNOD mice (n = 6). Data are pooled from two independent experiments and shown as mean ± SEM ***p<0.001 by unpaired t-test.

The inflammation in the liver was accompanied by fibrosis primarily localized to the portal tracts and central veins and with varying degrees of periportal and bridging fibrosis (Fig 2A) with deposits of matrix proteins such as collagen I (Fig 2B) and matrix metallopeptidase 9 (MMP9) (Fig 2C) and accumulation of anti-smooth muscle actin (ASMA) expressing cells were observed in the inflamed areas of the N-IF mouse liver (Fig 2D). The development of fibrosis was also biochemically confirmed by the significant increase in the level of hydroxyproline in livers from N-IF mice compared to asymptomatic 24αβNOD trangenic mice (Fig 2E). No obvious deterioration in the pathology of the liver nor any development of hepatocellular carcinoma was observed in N-IF mice up to 20 weeks of age or in a low number (n = 5) of N-IF mice that were followed for an extended time (age >35 weeks).

While the chronic inflammation and fibrosis was most evident in the liver, we also noted that several other organs including e.g the kidneys (S4A Fig) and skin (S4B Fig) were affected. Notably, the insulitis characteristic of NOD mice at this age was not observed in the N-IF mouse (S4C Fig).

### Transgenic NKT cells accumulating in inflammatory areas express type 2 inflammatory cytokines

**Fig 3:**
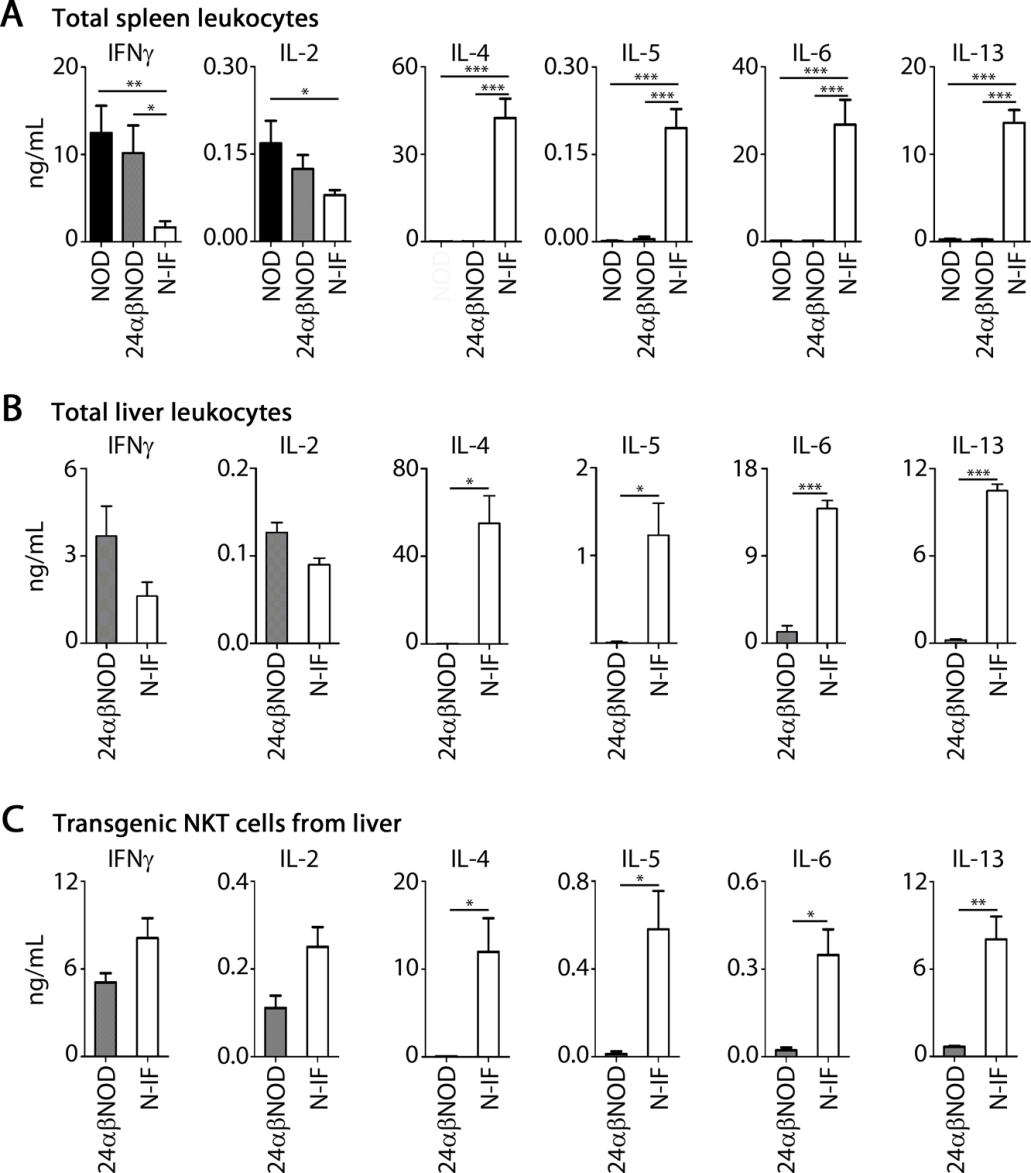
The inflammation in the N-IF mouse is characterized by a mixed expression of type 1 and type 2 cytokines. Cytokine levels (ng/mL) of IFNγ, IL-2, IL-4, IL-5, IL-6 and IL-13 in supernatant after 24h anti-CD3 activation of (A) total splenocytes from control NOD and 24αβNOD mice, and N-IF mice (n = 6). Data are pooled from two independent experiments and shown as mean ± SEM. *p<0.05, **p<0.01 and ***p<0.001 by unpaired *t*-test. (B) 24h anti-CD3 activation of total liver leukocytes from 24αβNOD and N-IF mice (n = 3). Data are from one experiment representative of two independent experiments. *p<0.05 and ***p<0.001 by unpaired *t*-test. (C) Cytokine concentration (ng/mL) in supernatant from CD3/CD28 activated liver Vα3.2^+^/Vβ9^+^ NKT cells sorted from 24αβNOD and N-IF mice (n = 3). Data are from one experiment representative of two independent experiments. Statistical analysis was performed using unpaired *t*-test where *p<0.05 and **p<0.01.

As illustrated in S5A Fig, the CD3^+^, transgenic NKT cells were found to be recruited to and accumulate in the liver overlapping the inflammatory areas described above. While the recruitment of various inflammatory cells to the liver of the N-IF mouse decreased the proportion of transgenic NKT cells compared to the 24αβNOD mouse (S5B Fig), the actual number of transgenic NKT cells in the liver did not significantly differ in the two mouse strains (S5C Fig). Because of the observed accumulation of transgenic NKT cells in the inflamed areas of the liver, we hypothesized that this cellular subset was driving the observed disease process. To address this issue we first compared the cytokine profile in the spleen of the N-IF mouse to the 24αβNOD control mouse and to the wild type NOD mouse. As expected, NOD spleen cells displayed high levels of IFNγ together with IL-2 but relatively low levels of type 2 cytokines such as IL-4 and IL-5 (Fig 3A). A similar cytokine profile was observed in the 24αβNOD mouse. In contrast, the cytokine profile of the N-IF mouse was altered with significantly reduced levels of IFNγ and IL-2 and increased expression of IL-4 and IL-5 but also production of additional type 2 cytokines like IL-13. In addition, we observed a dramatic increase in IL-6 production in the N-IF mouse. Similar to the systemic representation of cytokines in the N-IF mouse, the cytokine profile in total liver leukocytes (Fig 3B) as well as in the transgenic NKT cells isolated from N-IF livers (Fig 3C) displayed high levels of IL-6 together with the cytokines IL-4, IL-5 and IL-13 but with a retained expression of the cytokines IFNγ and IL-2. This data would indicate that the transgenic NKT cells in the N-IF mouse drive the inflammation and fibrosis development in the liver.

### The N-IF disease phenotype is controlled by the adaptive immune system

**Fig 4:**
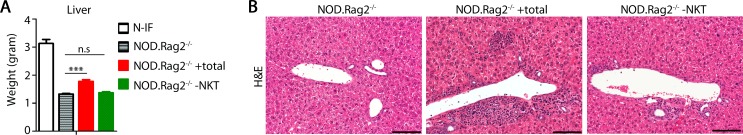
The N-IF mouse liver phenotype can be transferred by hematopoietic cells. (A) Liver weights of 10 week old N-IF mice, NOD.Rag2^-/-^ mice and NOD.Rag2^-/-^ mice adoptively transferred 5 weeks earlier with total spleen cells (NOD.Rag2^-/-^ +total, n = 7) or total spleen cells depleted of NKT cells (NOD.Rag2^-/-^ -NKT, n = 4) from 8–12 weeks old N-IF mice. Data are pooled from two independent experiments and shown as mean ± SEM. *** p<0.001by unpaired *t*-test. (B) Representative H&E stained liver sections from control NOD.Rag2^-/-^ mice and adoptively transferred NOD.Rag2^-/-^ mice receiving total spleen cells or total spleen cells depleted of transgenic NKT cells. Size bars are 100 μm.

**Fig 5:**
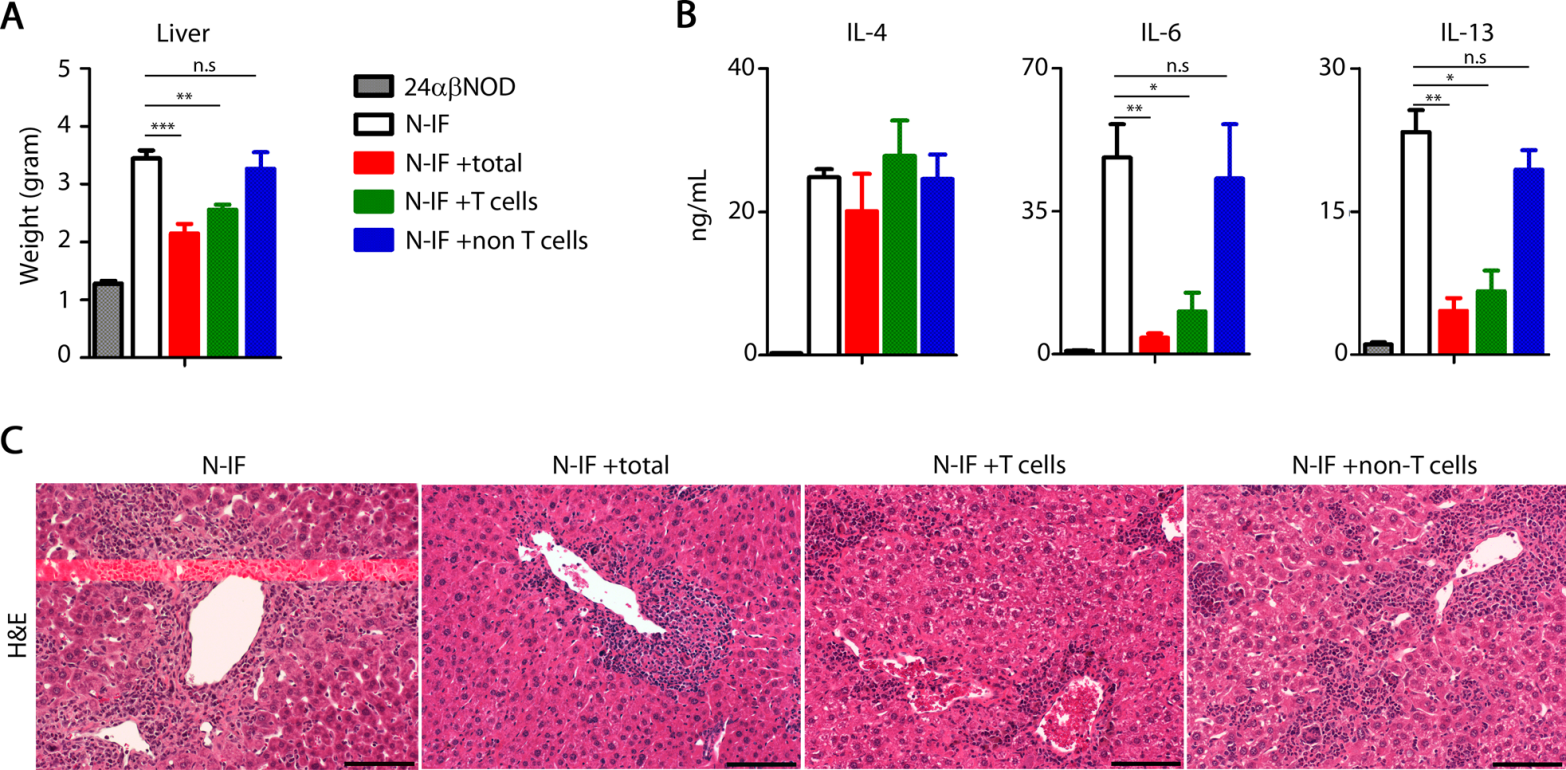
T cells reduce the N-IF mouse liver phenotype. (A) Liver weight of 10 week old 24αβNOD, N-IF or N-IF mice adoptively transferred 5 weeks earlier with spleen cells from 8–12 weeks old NOD mice: total spleen cells (total, n = 7), total spleen cells enriched for T cells (T cells, n = 4) or total spleen cells depleted of T cells (non-T cells, n = 4) Data are pooled from two independent experiments and shown as mean ± SEM. **p<0.01 and *** p<0.001by unpaired *t*-test. (B) Expression levels of IL-4, IL-6 and IL-13 (ng/mL) in supernatant from anti-CD3 activated total splenocytes from the same groups of mice as above. Data are pooled from two independent experiments with n = 4–7 per group and are shown as mean ± SEM. Statistical analysis was performed using unpaired *t*-test where *p<0.05 and **p<0.01. (C) Representative H&E stained livers from control N-IF mice and adoptively transferred N-IF mice receiving total spleen cells (total), total spleen cells enriched for T cells (T cells) or total spleen cells depleted of T cells (non T cells) from 8–12 weeks old NOD mice. Size bars are 100 μm.

We next tested the hypothesis that transgenic NKT cells were driving the disease development by transferring 25x10^6^ splenocytes from affected N-IF mice to 5 weeks old naive NOD.Rag2^-/-^ recipients. As illustrated in Fig 4, the transfer resulted in the development of liver disease after 5 weeks with a significant increase in liver weight accompanied by a histological picture of hepatobiliary disease and inflammation resembling that of the N-IF mouse (Figs 1B and 5). In contrast, when the majority of transgenic NKT cells were depleted (less than 0.5% Vα3.2^+^Vβ9^+^ cells remaining) from the N-IF splenocytes prior to transfer, the same disease inducing effect was not observed. We conclude, therefore, that the transgenic NKT cells generated in the immunodeficient N-IF mouse drive the inflammatory phenotype seen in the N-IF mouse.

Despite expressing the same transgenic TCRα and β chains as the N-IF mouse the 24αβNOD mouse did not develop the disease phenotype. This suggested that a RAG-dependent component of the adaptive immune system was able to suppress the disease promoting effect. To test this hypothesis, we adoptively transferred spleen cells derived from wild type, adult NOD mice to 5 weeks old N-IF mice at a stage when the recipient mice displayed a profound liver disease. In support of the hypothesis, total spleen cells, as well as spleen cells enriched for T cells, from wild type NOD mice were able to significantly reduce the inflammation phenotype 5 weeks post adoptive transfer (Fig 5). This was evidenced by reduced liver weights, reduced type 2 cytokine levels, albeit not IL-4, and less pronounced inflammation in the liver, compared to untreated N-IF mice. In contrast, no effect on the inflammation was observed when NOD spleen cells depleted of T cells were transferred to N-IF mouse recipients. In conclusion, the autoimmune effect of NKT cells can be controlled by the presence of other T cell clones.

### NOD genetic component(s) promotes liver disease in the N-IF mouse

**Fig 6:**
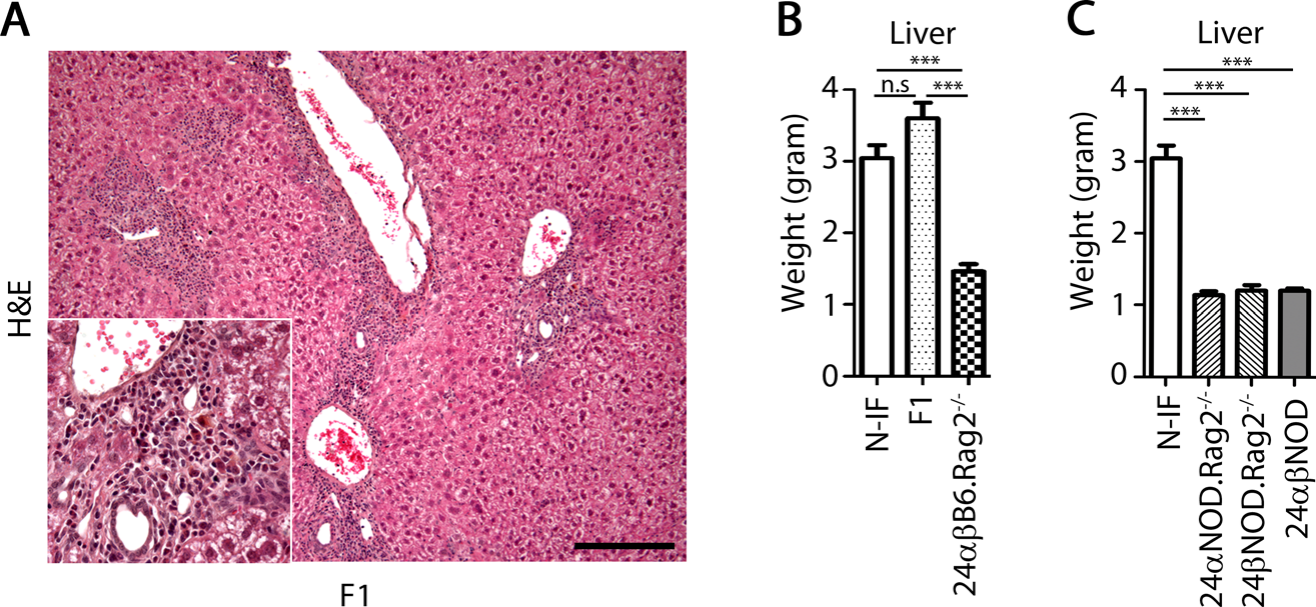
The N-IF mouse phenotype is dominant and dependent on a NOD gene(s). (A) H&E stained liver section from a 10 weeks old F1(24αβNOD Rag2^-/-^ x B6.Rag2^-/-^) mouse, with an inserted magnified image of the portal tract. Representative image from two independent experiments with a total of six mice is shown. The size bar is 100 μm. (B) Liver weight of 9–21 weeks old N-IF, F1(24αβNOD.Rag2^-/-^ x B6.Rag2^-/-^) and backcrossed 24αβB6.Rag2^-/-^ mice (n = 4–5). (C) Liver weight of 8–12 weeks old N-IF mice, single transgenic TCRα and TCRβ mice expressed in NOD.Rag2^-/-^ mice and 24αβNOD mice (n = 3–6). Data in B and C are from one experiment representative of two independent experiments and shown as mean ± SEM. Statistical analysis was performed using unpaired *t*-test where ***p<0.001.

Because the N-IF mouse model was established on the genetic background of the autoimmune prone NOD mouse, we reasoned that overlapping genetic factors could confer susceptibility to autoimmune diabetes in the NOD mouse and the inflammation/fibrosis developing in the N-IF mouse. To test this hypothesis, we crossed the N-IF mouse with B6.Rag2^-/-^ mice to generate F1(24αβNOD.Rag2^-/-^ x B6.Rag2^-/-^) mice. As illustrated in Fig 6A, these mice developed liver disease similar to the N-IF mouse suggesting that if such common susceptibility genes were present in the NOD genome, they acted in a dominant fashion. To confirm that the observed effect of the 24αβTCR transgene was dependent on the NOD genetic background, we next backcrossed the 24αβTCR transgenes of the N-IF mice onto the genetic background of the B6.Rag2^-/-^ mice generating 24αβB6.Rag2^-/-^ mice. As illustrated in Fig 6B, these mice did not develop the disease phenotype. Further, neither 24αNOD.Rag2^-/-^ nor 24βNOD.Rag2^-/-^ single transgenic mice developed disease (Fig 6C) suggesting that the disease phenotype of the N-IF mouse depended on the generation of the 24αβTCR transgenic cell population present in the N-IF mouse. In conclusion, the NOD genome contains one or more dominant gene(s) promoting the development of liver disease in the presence of a monoclonal population of transgenic NKT cells and in the absence of an endogenous adaptive immune system.

## Discussion

The N-IF mouse presented herein spontaneously develops liver inflammation and fibrosis associated with abnormal intrahepatic bile ducts. The development of fibrosis in this model is preceded by a state of chronic inflammation reflecting an important aspect characteristic of many human fibrotic disorders. Furthermore the N-IF mouse provides several previously unmet demands on an animal model for fibrosis e.g. in terms of reproducibility and spontaneous onset compared with most presently available animal models for fibrosis. The N-IF mouse was originally generated to investigate the role of type II NKT cells as regulators of the autoimmune diabetes developing in the NOD mouse. We previously reported that transgenic expression of the 24αβTCR resulted in the production of type II NKT cells, efficiently inhibiting the development of diabetes in the NOD mouse [16, 18]. In view of this finding, it was unexpected that expression of the same transgenic TCR in an immunodeficient NOD.Rag2^-/-^ genetic background resulted in the development of progressive inflammation and fibrosis in multiple organs.

To elucidate the mechanisms underlying this process we carried out adoptive transfer experiments demonstrating that splenocytes from the N-IF mouse could induce inflammation in naïve NOD.Rag2^-/-^ recipients while splenocytes depleted from transgenic NKT cells could not (Fig 4). Thus, the pathology of the N-IF mouse is driven by the transgenic NKT cells expressing a type II NKT cell TCR made up by the Vα3.2 and the Vβ9 TCR chains. Further, the failure to develop the inflammatory and fibrotic liver disease of the single transgenic 24αNOD.Rag2^-/-^ and 24βNOD.Rag2^-/-^ mice provided evidence that the formation of a complete TCR and thus the generation of a functional NKT cell population was required for the disease to develop. While the full understanding of the underlying molecular mechanisms still remains to be elucidated, we noted that the monoclonal population of transgene expressing NKT cells of the N-IF mouse shifted from the predominant expression of IFNγ and IL-2 in the 24αβNOD mice to a mixed type 1/type 2 cytokine pattern with high expression also of IL-4, IL-5, IL-13 and IL-6. This cytokine profile is likely to underlie the activation and recruitment of inflammatory cells including neutrophils, eosinophils and mast cells to the liver. Further, the observed matrix deposition and accumulation of ASMA positive cells in the inflamed areas of the liver, together with the unaltered AST/ALT ratio, is in line with the notion of a crosstalk between the transgenic NKT cells and hepatic stellate cells as a major mechanism leading to the development of extensive periportal and bridging fibrosis in the N-IF mouse.

The two major subsets of NKT cells, type I and type II, have been assigned opposing roles in chronic liver disease [19, 20] where Type I NKT cells have been reported to have mainly a pro-inflammatory role while Type II NKT cells have been suggested to have a potentially regulating role [21]. In this scenario, the pro-inflammatory and pro-fibrotic properties of the transgenic NKT cell population of the N-IF mouse may be counterintuitive, given that the transgenic TCR was derived from a Type II NKT cell [16]. While further studies will be required to provide a full understanding of this observation, the fact that the N-IF transgenic NKT cells express a mixed Th1/Th2 cytokine profile may provide a clue to their disease promoting effect.

The disease phenotype of the N-IF mouse can efficiently be inhibited by a functional adaptive immune system as exemplified by the absence of inflammation and fibrosis in the 24αβNOD transgenic mouse. Under these conditions, the transgene-expressing NKT cell population as well as the systemic cytokine profile is dominated by IFNγ rather than type 2 cytokines. It is interesting to note that despite the reduction in inflammation resulting from the transfer of NOD T cells, as well as the restoration of the expression levels of most pro-inflammatory cytokines, no obvious reduction in IL-4 production was observed. This suggests that at this stage of the disease process, IL-4 cannot be the main driving force of the inflammation. The fact that T cells from wild type NOD mice could, at least in part, revert the type 2 biased cytokine profile suggests that this shift is controlled by a so far unidentified T cell component(s). Regulatory T cells (Treg) have a critical role in regulating immune mediated liver disease [22]. Thus, Treg cells constitute a plausible candidate for mediating the observed control of the N-IF mouse phenotype and this hypothesis is presently being addressed.

The failure of 24αβB6.Rag2^-/-^ mice to develop disease demonstrated that the NOD mouse contained disease promoting genetic factors(s). Moreover the genetic crossing of the N-IF mouse to B6.Rag2^-/-^ mice to generate F1(24αβNOD.Rag2^-/-^ x B6.Rag2^-/-^) mice expressing the 24αβTCR revealed that the NOD gene(s) promoting the disease development were dominant. Genetic dissection of the non-obese diabetic (NOD) mouse has revealed that the NOD genetic background promotes the development of different but potentially related autoimmune disease phenotypes when combined with different specific genetic components [23–25]. The inflammatory phenotype found in the N-IF mouse liver resembles some aspects of the pathology associated with primary biliary cirrhosis (PBC) [26]. Interestingly, two NOD congenic strains, NOD.c3c4 [24, 27] and NOD.ABD [28], have both previously been reported to develop biliary disease. The occurrence of biliary disease, albeit with differences in their characteristics, in these three different mouse strains sharing a NOD genetic background is striking and supports the notion that the NOD genome contains susceptibility genes common to autoimmune diabetes and biliary disease [29]. The fibrosis observed in the portal tracts of the N-IF mouse is of particular interest since it is typical of human PBC but not represented in most other models of the disease [11, 29]. Genetic mapping of the N-IF genome would give further insight into which NOD genetic components that are of importance for the liver inflammation and fibrosis development in the N-IF mouse.

In summary, we have established a new mouse model that spontaneously develops progressive inflammation and fibrosis in multiple organs that provides a complement to existing animal models of fibrotic conditions. In addition to offering a new and unique model for effect tests of anti-fibrotic and anti-inflammation therapeutic regimes, the N-IF model enables novel approaches to elucidate the cellular and molecular mechanisms underlying the pathogenesis of these disease conditions.

## Supporting Information

S1 Fig Gating strategy for transgenic NKT cells and eosinophilic/neutrophilic granulocytes in liver. Doublets are excluded using strict SSC-A/SSC-H and FSC-A/FSC-H gates. Leukocytes are defined by the SSC/FSC gate as well as by the expression of CD45. The transgenic NKT cells are defined by its T cell receptor (Vα3.2/Vβ9) and eosinophilic/neutrophilic granulocytes by the CD11b expression along with SiglecF (for eosinophils) and Ly6G (for neutrophils).
(TIF)

S2 Fig The N-IF mouse develops hepatomegaly and chronic inflammation. (A) Gross pathology of livers from the control 24αβNOD mouse (left) and the N-IF mouse (right) at 8 weeks of age. (B) Representative toluidine blue (TolB) stained liver sections from 12 weeks old N-IF mice and control 24αβNOD mice. White arrow heads show mast cells. (C) H&E stained liver sections from 12 weeks old N-IF mice. Black arrow indicates multinucleated giant cell and arrow head show megakaryocyte. (D) Extramedullary hematopoiesis in the liver of 12 weeks old N-IF mice. Scale bars are 50 μm.
(TIF)

S3 Fig The N-IF mouse display liver inflammation at an early age. Representative H&E stained liver sections from 3 weeks old N-IF mice (top) and 24αβNOD mice (bottom). The scale bars are 200 μm in the overview images and 50 μm in the enlarged images.
(TIF)

S4 Fig The N-IF mouse display renal glomerular collagen deposits and inflammation in the skin. (A) Masson’s Trichrome stained kidney sections showing collagen deposits in blue, and (B) H&E stained sections of the ear from 10 weeks old N-IF mice and 24αβNOD control mice. Scale bars are 50 μm for the kidney sections and 100 μm for the ear sections. (C) Representative pancreas section from a total of seven 16 weeks old N-IF mice stained with H&E. Scale bar is 200 μm.
(TIFF)

S5 Fig Transgenic NKT cells accumulate in the portal tract in the N-IF mouse liver. (A) Fluorescence images showing the liver portal area of 8 weeks old N-IF and control 24αβNOD mice stained with CD3 (red) and DAPI (blue). Representative images from two independent experiments with a total of six mice are shown. Scale bars are 100 μm. (B) Flow cytometry analyses of liver Vα3.2/Vβ9 positive NKT cells gated from viable CD45+ cells. Representative dot-plots from three independent experiments with a total of nine mice are shown. (C) Total number of NKT cells in liver from N-IF and 24αβNOD mice (n = 9–12). Data are pooled from three independent experiments and shown as mean ± SEM. Statistical analysis was performed using unpaired t-test.
(TIF)

S1 Table Serum levels of liver markers- Sex and age matched N-IF (n = 10) and 24αβNOD control mice were bled and serum was collected and sent to The University Animal Hospital, SLU, Uppsala for measuring AST, ALT, ALP, total bilirubin and Bile acid in serum using a fully automated Architect c4000 (Abbott Laboratories, Abbott Park, IL, US). Statistical analysis was done by unpaired *t*-test and shown as mean ± SEM. ns = non significant. p values < 0.5 are indicated.
(DOC)
