# Supplementary figures and images for: A New Mouse Model That Spontaneously Develops Chronic Liver Inflammation and Fibrosis
Source: PLoS One. 2016 Jul 21;11(7):e0159850. doi: 10.1371/journal.pone.0159850 (PMC4956255; doi:10.1371/journal.pone.0159850)

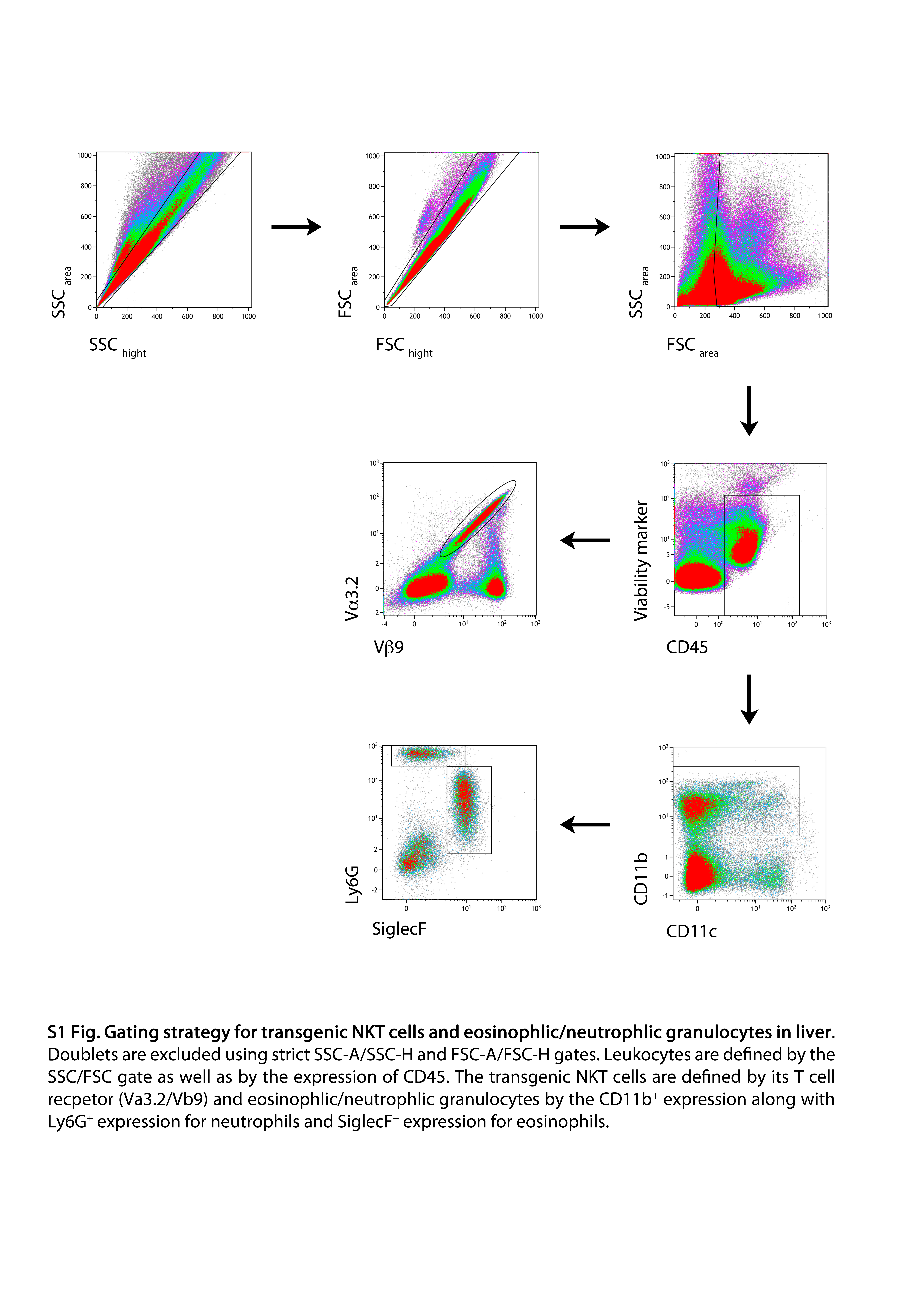

Supplement: S1 Fig — Doublets are excluded using strict SSC-A/SSC-H and FSC-A/FSC-H gates. Leukocytes are defined by the SSC/FSC gate as well as by the expression of CD45. The transgenic NKT cells are defined by its T cell receptor (Vα3.2/Vβ9) and eosinophilic/neutrophilic granulocytes by the CD11b expression along with SiglecF (for eosinophils) and Ly6G (for neutrophils). (TIF) [file pone.0159850.s001.tif]

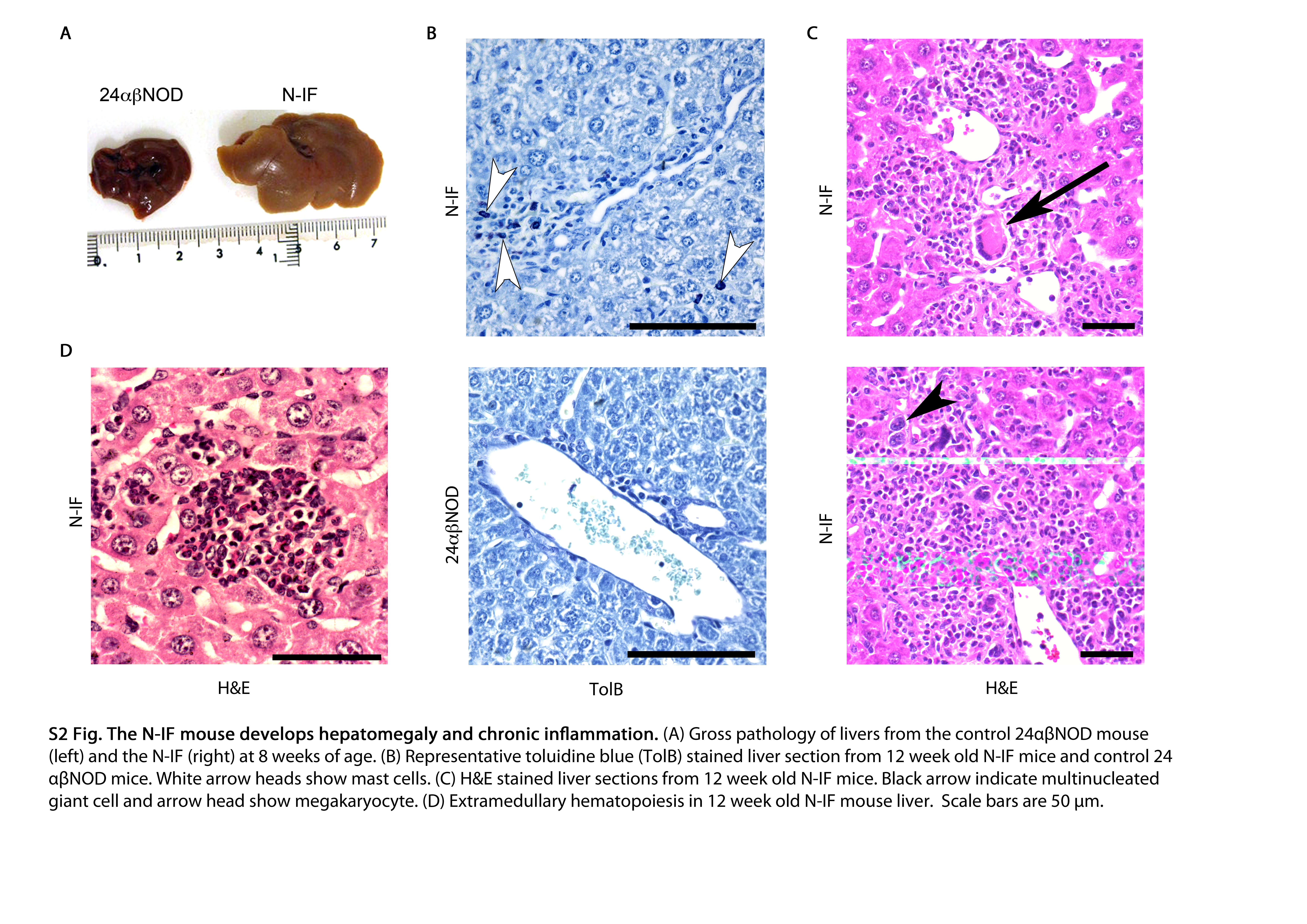

Supplement: S2 Fig — (A) Gross pathology of livers from the control 24αβNOD mouse (left) and the N-IF mouse (right) at 8 weeks of age. (B) Representative toluidine blue (TolB) stained liver sections from 12 weeks old N-IF mice and control 24αβNOD mice. White arrow heads show mast cells. (C) H&E stained liver sections from 12 weeks old N-IF mice. Black arrow indicates multinucleated giant cell and arrow head show megakaryocyte. (D) Extramedullary hematopoiesis in the liver of 12 weeks old N-IF mice. Scale bars are 50 μm. (TIF) [file pone.0159850.s002.tif]

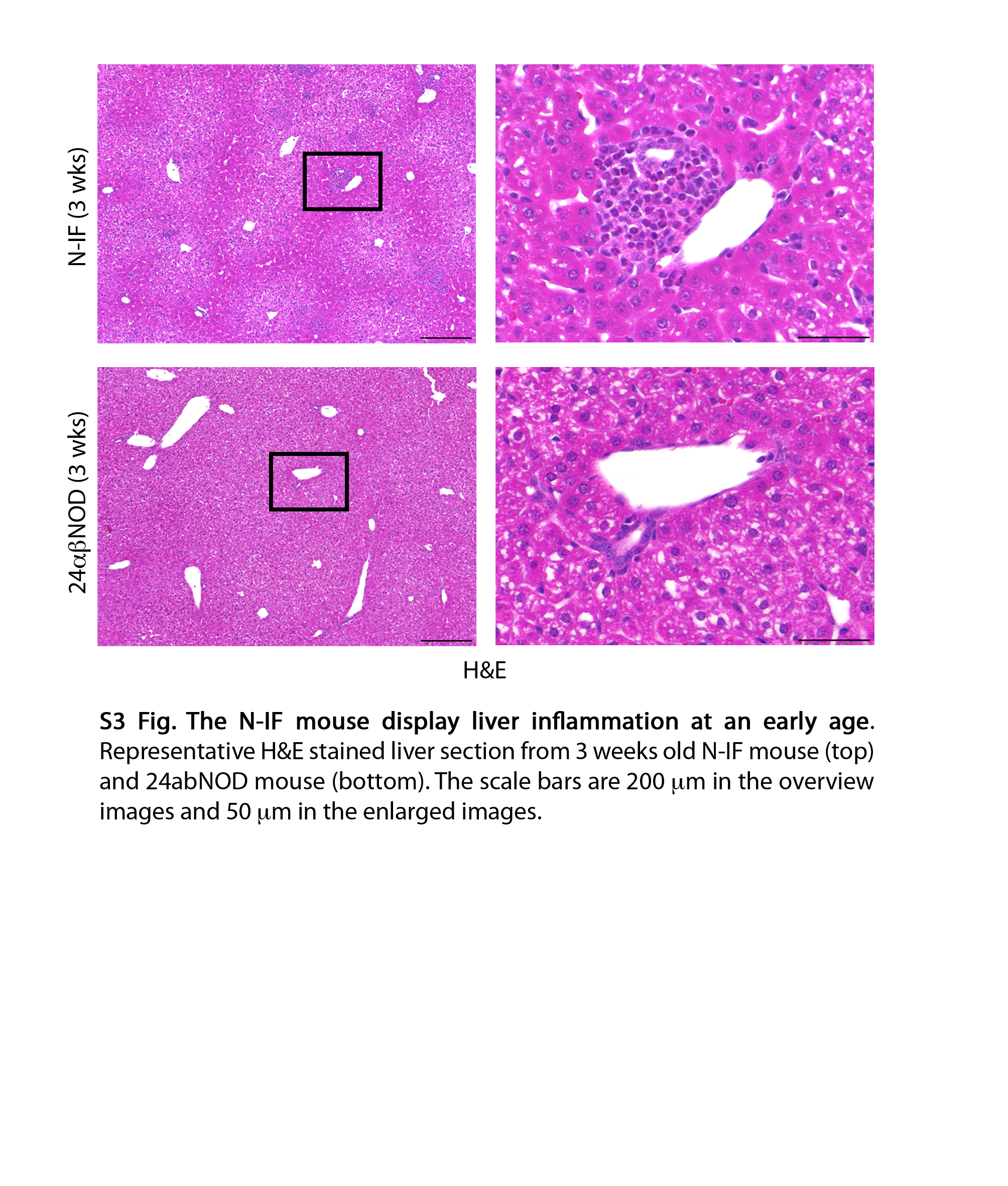

Supplement: S3 Fig — Representative H&E stained liver sections from 3 weeks old N-IF mice (top) and 24αβNOD mice (bottom). The scale bars are 200 μm in the overview images and 50 μm in the enlarged images. (TIF) [file pone.0159850.s003.tif]

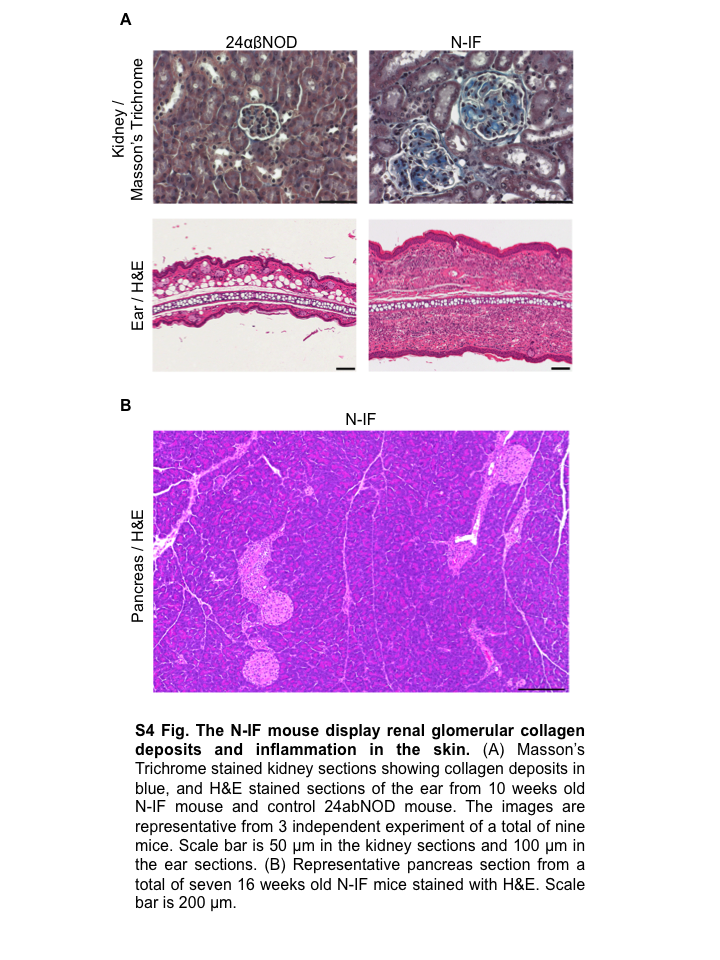

Supplement: S4 Fig — (A) Masson’s Trichrome stained kidney sections showing collagen deposits in blue, and (B) H&E stained sections of the ear from 10 weeks old N-IF mice and 24αβNOD control mice. Scale bars are 50 μm for the kidney sections and 100 μm for the ear sections. (C) Representative pancreas section from a total of seven 16 weeks old N-IF mice stained with H&E. Scale bar is 200 μm. (TIFF) [file pone.0159850.s004.tiff]

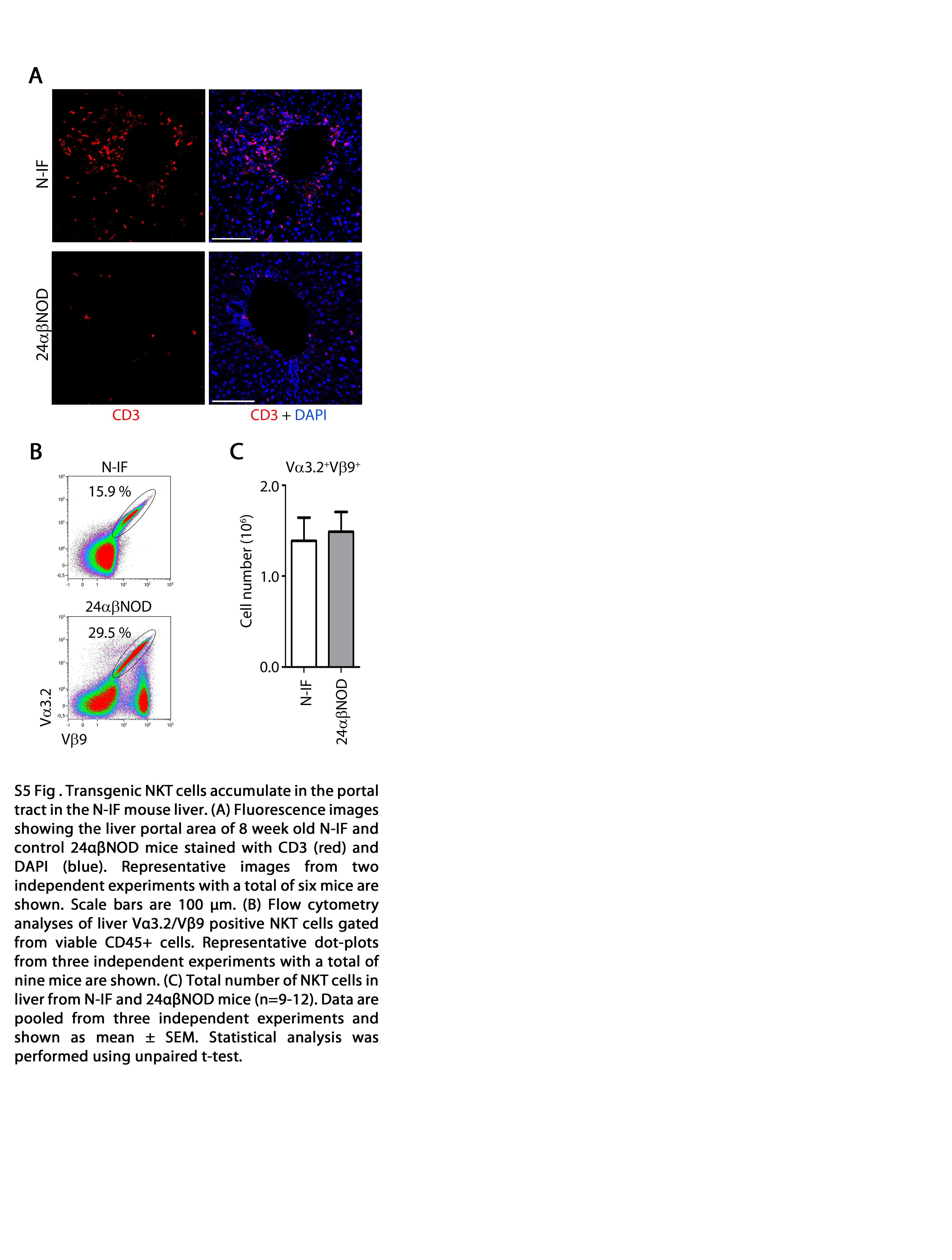

Supplement: S5 Fig — (A) Fluorescence images showing the liver portal area of 8 weeks old N-IF and control 24αβNOD mice stained with CD3 (red) and DAPI (blue). Representative images from two independent experiments with a total of six mice are shown. Scale bars are 100 μm. (B) Flow cytometry analyses of liver Vα3.2/Vβ9 positive NKT cells gated from viable CD45+ cells. Representative dot-plots from three independent experiments with a total of nine mice are shown. (C) Total number of NKT cells in liver from N-IF and 24αβNOD mice (n = 9–12). Data are pooled from three independent experiments and shown as mean ± SEM. Statistical analysis was performed using unpaired t-test. (TIF) [file pone.0159850.s005.tif]
