# Supplementary material for: A New Mouse Model That Spontaneously Develops Chronic Liver Inflammation and Fibrosis
Source: PLoS One. 2016 Jul 21;11(7):e0159850. doi: 10.1371/journal.pone.0159850 (PMC4956255; doi:10.1371/journal.pone.0159850)
Supplement: S1 Table — Statistical analysis was done by unpaired t-test and shown as mean ± SEM. ns = non significant. p values < 0.5 are indicated. (DOC) [file pone.0159850.s006.doc]

**S1 Table. Serum levels of liver markers-** Sex and age matched N-IF (n=10) and 24αβNOD control mice were bled and serum was collected and sent to The University Animal Hospital, SLU, Uppsala for measuring AST, ALT, ALP, total bilirubin and Bile acid in serum using a fully automated Architect c4000 (Abbott Laboratories, Abbott Park, IL, US). Statistical analysis was done by unpaired *t*-test and shown as mean ± SEM. ns= non significant. p values < 0.5 are indicated.

| **Mice** | **Age** | **n** | **AST IU/L**  **(SEM)** | | **ALT IU/L**  **(±SEM)** | **AST/ALT** | **Bilirubin tot**  **(±SEM)** | **ALP IU/L**  **(±SEM)** | **Bile acids μmol/L** |
| --- | --- | --- | --- | --- | --- | --- | --- | --- | --- |
| **24αβNOD** | 8 w | 10 | 99.9  (±7.59) | 15.00  (±1.09) | | 6.82  (±0.54) | 2.90  (±0.18) | 86.70  (±6.06) | 2.02  (±0.32) |
| **NIF** | 8 w | 10 | 81.90  (±8.32)  ns | 15.90  (±3.13)  ns | | 5.77  (±0.53)  ns | 2.96  (±0.15)  ns | 34.70  (±1.98)  **p<0.0001** | 5.26  (±1.09)  **p=0.0031** |
